# Survival in patients with surgically treated brain metastases: does infratentorial location matter?

**DOI:** 10.1007/s10143-023-01986-6

**Published:** 2023-03-30

**Authors:** Motaz Hamed, Anna-Laura Potthoff, Muriel Heimann, Niklas Schäfer, Valeri Borger, Alexander Radbruch, Ulrich Herrlinger, Hartmut Vatter, Matthias Schneider

**Affiliations:** 1 Department of Neurosurgery, University Hospital Bonn, Venusberg-Campus 1, 53127 Bonn, Germany; 2 Division of Clinical Neuro-Oncology, Department of Neurology, University Hospital Bonn, Venusberg-Campus 1, 53127 Bonn, Germany; 3 Department of Neuroradiology, University Hospital Bonn, Bonn, Germany

**Keywords:** Surgery for brain metastasis, Infra- versus supratentorial BM location, Survival

## Abstract

Surgical resection is a common treatment modality for brain metastasis (BM). Location of the BM might significantly impact patient survival and therefore might be considered in clinical decision making and patient counseling. In the present study, the authors analyzed infra- and supratentorial BM location for a potential prognostic difference. Between 2013 and 2019, 245 patients with solitary BM received BM resection at the authors’ neuro-oncological center. In order to produce a covariate balance for commonly-known prognostic variables (tumor entity, age, preoperative Karnofsky Performance Score, and preoperative Charlson Comorbidity Index), a propensity score matching at a ratio of 1:1 between the cohort of patients with infra- and supratentorial BM location was performed using R. Overall survival (OS) rates were assessed for both matched cohorts of patients with BM. Sixty-one of 245 patients (25%) with solitary BM exhibited an infratentorial tumor location; 184 patients (75%) suffered from supratentorial solitary BM. Patients with infratentorial BM revealed a median OS of 11 months (95% confidence interval (CI) 7.4–14.6 months). Compared with this, median OS for the group of 61 individually matched patients with solitary supratentorial solitary BM was 13 months (95% CI 10.9-15.1 months) (*p* = 0.32). The present study suggests that the prognostic value of infra- and supratentorial BMs does not significantly differ in patients that undergo surgery for solitary BM. These results might encourage physicians to induce surgical therapy of supra- and infratentorial BM in a similar manner.

## Introduction

Improved and individualized cancer therapy is leading to a growing number of patients with varying malignancies experiencing metastases to the brain in the course of their disease [1]. The occurrence of brain metastases (BM) represents an important (prognostic) stage in the progression of systemic cancer [2]. In patients with BM, neurosurgical resection often constitutes a key pillar of treatment [3]. Despite a myriad of treatment adaptations/optimizations due to ever new findings in the field of different malignancies, early surgical/radiotherapy treatment of BM is consistently deemed crucial with respect to the prognosis of advanced cancer [4]. Surgical therapy is often considered for solitary/larger BM and requires individualized risk/benefit consideration against non-invasive treatment modalities but might nonetheless offer a significant survival benefit [5].

While BM causing early symptoms can be treated at an early stage, they are often located in eloquent areas [5]. This eloquent location might facilitate early symptom emergence, but it also impairs surgical resectability [5]. Here, BM of the posterior fossa might be a peculiarity as their space-occupying effect often leads to early symptoms, and yet they are well-resectable. Even though BM location is not included in commonly used methods of predicting patient outcomes, clinicians still consider it important and often make decisions about local treatment based on where the BM is located [6]. According to published studies, some researchers strongly believe that BMs located in the posterior fossa are associated with worse prognosis and can lead to neurological disability through brainstem damage, hydrocephalus, and herniation of the cerebellum [7].

Therefore, the present study is intended to focus on the prognostic influence of the location in the posterior fossa and to provide clinical data for this important entity.

## Methods

All patients who had undergone surgery for solitary BM between 2013 and 2019 at the authors’ neuro-oncological university center were entered into a computerized database. Patients with multiple BM and/or leptomeningeal involvement were excluded. The study was conducted in accordance with the Declaration of Helsinki, and the protocol was approved by the Ethics Committee of the University Hospital Bonn (No. 250/19). Informed consent was not sought as a retrospective study design was chosen.

Patient characteristics surveyed for further analysis consisted of radiological features, preoperative laboratory values, and BM location, as well as the location of the primary malignancy, the preoperative functional status of the affected patient, and the presence of other systemic metastases. In terms of location, two groups were established for further investigation on the basis of the supra- or infratentorial location of the BM. Infratentorial location was defined as within the cerebellum, and brainstem lesions were excluded from analysis. The functional status of patients undergoing surgery was assessed preoperatively according to the Karnofsky Performance Score (KPS) [8]. The Charlson Comorbidity Index (CCI) was used to evaluate the comorbidity burden of patients prior to surgery as previously described [3]. During a weekly tumor board meeting, all the treatment strategies applied and further investigated were determined individually for each individual patient by interdisciplinary consensus and, if necessary, coordinated with the referring physicians and/or taken into account previous oncological therapies [9]. In the present study, only patients with indication for surgical therapy were considered and further analyzed resulting in a consecutive cohort of patients with surgically treated BM. After BM resection, patients are transferred to their transferring hospital in order to conduct postoperative oncological treatment [10].

To evaluate perioperative complication profiles in patients undergoing BM resection, a list of adverse events known as patient safety indicators (PSIs) and hospital-acquired conditions (HACs) was used as previously described [11]. These events were established by the Agency for Healthcare Research and Quality and the Center for Medicare and Medicaid Services. The PSIs included occurrences such as pressure ulcers, transfusion reactions, and postoperative hemorrhage, while HACs included screening for pneumonia, fall injuries, and catheter-associated urinary tract infections. Specific to cranial surgeries, complications such as cerebrospinal fluid leakage and postoperative seizures, were classified as cranial-surgery-related complications (CSCs) as previously described [12]. Any intra- or postoperative adverse events that occurred within 30 days of the initial resection, with or without further surgical interventions, were considered perioperative complications.

### Matching procedure

Matching was used to control for measured pre-treatment variables that are prognostic of the outcome. For the matched-pair analysis, the statistical computing program R (version 4.1.2; The R Foundation for Statistical Computing, https://www.r-project.org/) was used as previously described [11]. A propensity score matching was performed at a ratio of 1:1 between the cohort of 61 patients with infratentorial BM and a cohort of 184 patients with supratentorial BM. To produce a covariate balance in the two groups and therefore increase the robustness of the data, the following known prognostic parameters were selected for matching: age, KPS, and CCI at admission and tumor entity. The balance was measured by the standardized mean differences, variance ratios, and empirical cumulative density function statistics and visualized using a Love plot.

### Statistical analysis

Data analyses were performed using the SPSS computer software package (version 27, IBM Corp., Armonk, NY). Categorical variables were analyzed in contingency tables using the Fisher’s exact test in case of only two variables. If more than two variables had to be analyzed, the chi-square test was applied. The Mann-Whitney *U*-test was chosen to compare continuous variables as data were mostly not normally distributed. OS was analyzed by the Kaplan-Meier method. The Gehan-Breslow-Wilcoxon test was used to compare survival rates in case of supratentorial and infratentorial BMs. Results with *p* < 0.05 were considered statistically significant.

## Results

### Patient and tumor characteristics

Between 01/2013 and 01/2019, 395 patients had undergone resection of BM at the neuro-oncological center of the University Hospital Bonn. Thirty-seven patients were excluded from further analysis due to the lack of sufficient follow-up information. One hundred thirteen of the remaining 358 patients (32%) revealed multiple BM at admission, and 245 of 358 patients (68%) suffered from solitary BM. Mean age of the patients with solitary BM was 64 years (SD +/- 12 years) with 122 female (50%) and 123 male patients (50%). Most commonly BM originated from lung cancer (*n* = 100, 41%), followed by breast cancer (*n* = 29, 12%) and melanoma (*n* = 28, 11%). Median preoperative KPS for the entire patient cohort was 80 (IQR 70–90). Sixty-one of 245 patients (25%) suffered from infratentorial BM; 184 of 245 patients (75%) exhibited supratentorial BM. One hundred twenty-six patients (51%) died within 1 year after BM resection. mOS for the entire study cohort was 13 months (95% CI 10.4–16.6). Further details are given in Table 1.

**Table 1:** Baseline characteristics (values represent number of patients unless indicated otherwise (%))

	*n* = 245
Mean age (yrs, ± SD)	64 ± 12
Female sex	122 (50)
Primary site of cancer	
NSCLC	100 (41)
Breast	29 (12)
Melanoma	28 (11)
Others	88 (36)
Preoperative KPS (IQR)	80 (70–90)
Preoperative median CCI (IQR)	11 (10–12)
Median OP duration (min, IQR)	165 (140–210)
Infratentorial BM location	61 (25)
Supratentorial BM location	184 (75)
1-Year mortality	126 (51)
mOS (mo, 95% CI)	15 (11.4-18.6)

BM, brain metastasis; CCI, Charlson Comorbidity Index; CI, confidence interval; IQR, interquartile range; KPS, Karnofsky Performance Score; mo, months; min, minutes; mOS, median overall survival; NSCLC, non-small cell lung carcinoma; OP, operation; SD, standard deviation; yrs, years

### Comparative matched pair survival analysis for infra- and supratentorial BM location

**Fig. 1:**
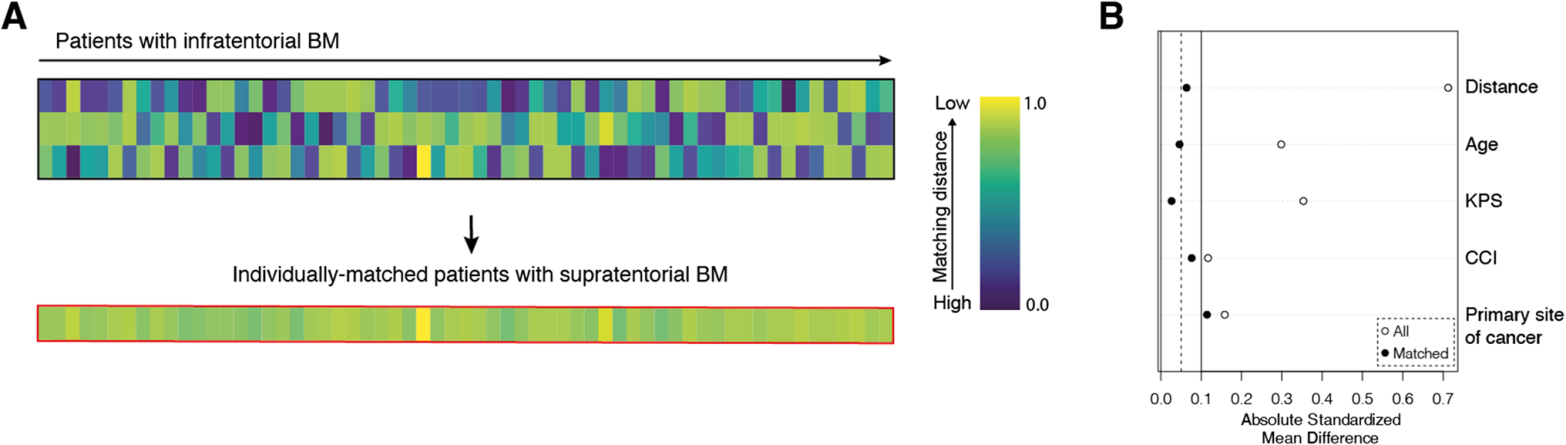
Illustration of the matching procedure for patients with surgically treated BM dependent on infra- versus supratentorial location of the BM. **A** Comparative matched pair analysis at a ratio of 1:1 identifies 61 out of 184 patients with supratentorial BM that individually correspond to the present series of 61 patients with infratentorial BM. Heat map as color-coded illustration of the matching strategy of patients with supratentorial BM to individually matched infratentorial cases by means of age at admission, KPS at admission, CCI at admission, and tumor entity as matching parameters. **B** Love plot depicting the balance of the matching analysis for each matching parameter determined by the standardized mean differences. BM, brain metastasis; CCI, Charlson Comorbidity Index; KPS, Karnofsky Performance Score

**Fig. 2:**
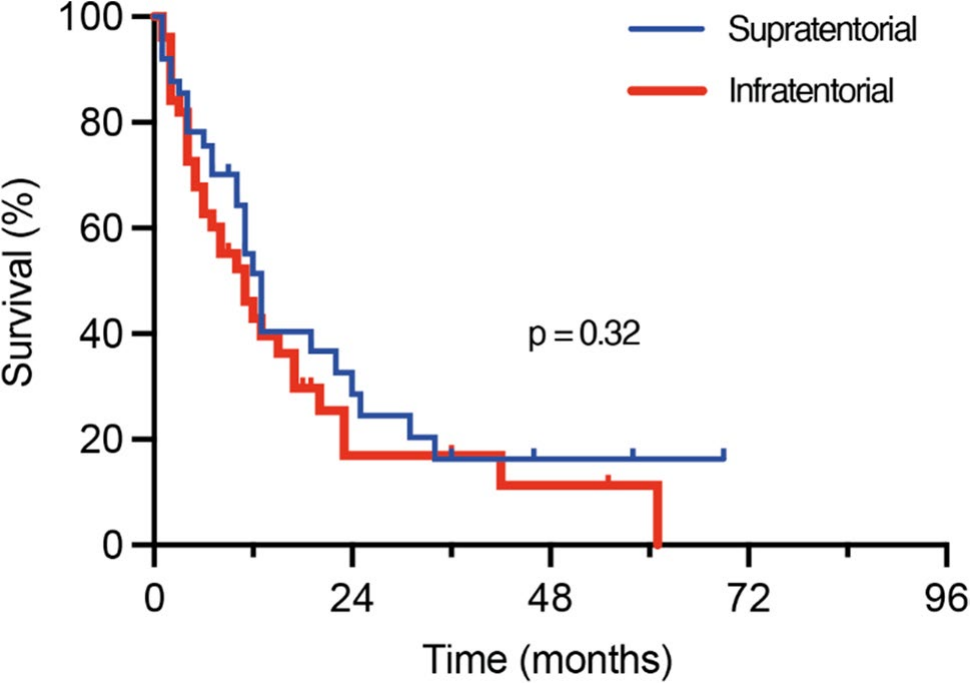
Survival rates of patients with supratentorial and infratentorial BMs do not differ. Kaplan-Meier curves for OS of patients with surgically treated BM stratified into supra- versus infratentorial location of the resected BM. BM, brain metastasis; OS, overall survival

Comparative analysis of age, sex, preoperative KPS, and CCI as well as the presence of extracranial metastasis at the time of BM diagnosis revealed a homogeneous distribution between the groups of synchronous and metachronous BM occurrence (Table 2, Fig. 1). In order to compare survival rates of patients with surgically treated BM dependent on infra- and supratentorial tumor location, a multivariate and propensity score matching with additional balance optimization was performed. Patients with infratentorial BM location were individually matched at a ratio of 1:1 to a cohort of 184 patients with supratentorial BM location that had undergone resection of BM between 2013 and 2018 at our neuro-oncological center (Fig. 1). Patient age, preoperative KPS, preoperative CCI, and primary site of cancer as known prognostic parameters for survival in patients with BM were chosen as matching variables. The matched-pair analysis yielded two individually matched cohorts of 61 patients with infratentorial and 61 patients with supratentorial solitary BM that did not significantly differ with regard to abovementioned prognostic survival parameters (Table 2). Fifty-two of 61 patients with supratentorial BM (85%) received adjuvant radiation therapy compared to 56 of 61 patients with infratentorial BM (92%) (*p* = 0.39). The respective data for postoperative chemo- and/or immunotherapy were 49 of 61 patients for supratentorial (80%) and 45 of 61 patients with infratentorial BM (74%) (*p* = 0.51) (Table 2). One-year mortality did not significantly differ between these groups (39 patients infratentorial vs. 37 patients supratentorial, *p* = 0.71) (Table 2). Similarly, mOS did not significantly differ between the two patient groups of differing BM locations (infratentorial 11 months (95% CI 7.4–14.6) vs. supratentorial 13 months (95% CI 10.9–15.1), *p* = 0.32) (Table 2, Fig. 2).

**Table 2:** Comparative matched pair analysis dependent on supra- and infratentorial BM location (values represent number of patients unless indicated otherwise (%))

	Suptratentorial location *n* = 61	Infratentorial location *n* = 61	*p* Value
Matching variables			
Median age (yrs, ± SD)	67 ± 10	66 ± 12	0.66
Primary site of cancer			
NSCLC	28 (46)	25 (41)	0.59
Breast	5 (8)	11 (18)	0.11
Melanoma	8 (13)	4 (7)	0.22
Others	20 (33)	21 (34)	0.85
Median KPS (IQR)	80 (70–90)	80 (70–90)	0.82
Median CCI (IQR)	11 (10–12)	10 (10–11)	0.51
Outcome variables			
Female sex	25 (41)	31 (51)	0.37
ASA ≥ 3	40 (66)	38 (62)	0.85
Median OP duration (min, IQR)	177 (143–214)	166 (146–200)	0.3
Perioperative complications			0.95
HACs	1 (2)	1 (2)	1.0
PSIs	1 (2)	1 (2)	1.0
CSCs	1 (2)	2 (3)	0.56
Adjuvant treatment			
Radiation therapy	52 (85)	56 (92)	0.39
Chemo-/immunotherapy	49 (80)	45 (74)	0.51
1-Year mortality	37 (61)	39 (64)	0.71
mOS (mo, 95% CI)	13 (10.9–15.1)	11 (7.4–14.6)	0.32

ASA, American society of anesthesiologists; BM, brain metastasis; CCI, Charlson Comorbidity Index; CI, confidence interval; CSCs, cranial surgery-related complications; HACs, hospital-acquired conditions; IQR, interquartile range; KPS, Karnofsky Performance Score; mo, months; min, minutes; mOS, median overall survival; NSCLC, non-small cell lung carcinoma; OP, operation; PSIs, patient safety indicators; SD, standard deviation; yrs, years

## Discussion

The current study examined survival outcomes in patients who had undergone surgery for BM, either in the supra- or infratentorial region. Applying a covariate balance for known prognostic parameters in surgery for BM, a propensity score matching between patients with supra- and infratentorial BM location showed that there were no significant differences in survival between these two groups.

The mOS for the entire study cohort in the current study was 15 months. For survival comparison with other studies on patients with BM, it is important to note that the patient cohort in the present study only included patients who had undergone resection surgery. This concept is illustrated in a study of 708 patients, where the location of cerebellar brain metastases (BM) was initially associated with poorer survival when all patients were included in the analysis [13]. However, when the analysis was restricted to patients with resection surgery, there was no significant difference in survival between patients with cerebellar BMs and those with BMs in other locations [13]. Further, as multiple BM are known to be an important negative prognostic predictor of survival following BM surgery [3, 14], the current study focused specifically on solitary BM. In comparison to several studies suggesting infratentorial BM location to be associated with worsened survival outcome parameters [15, 16], the present study made use of a propensity score matching in order to ensure a homogeneous distribution of age [17], tumor entity [14], preoperative KPS [14, 18] and comorbidity burden [3] as known important prognostic parameters in patients with BM. One further factor that may contribute to the variability in research findings on the prognostic value of infra- versus supratentorial BM location might be reasoned in undifferentiated analyses of both cerebellar and brainstem lesions. While brainstem tumors are often not able to be surgically removed and the dose of radiation used to treat them may be limited due to the risk of side effects, tumors located in the cerebellum are often surgically removable and can be effectively treated with either postoperative radiation or definitive radiosurgery [6]. When studies combine brainstem and cerebellar tumors into a single category (infratentorial location), it can be challenging to accurately interpret the results and understand the specific impact of tumor location on patient outcomes. In a study by Trifiletti et al., the survival outcomes of patients with brainstem tumors who were treated with stereotactic radiosurgery (SRS) were compared to a group of patients with non-brainstem metastases who were also treated with SRS [19]. The authors found that the median survival of patients with brainstem tumors was significantly shorter than that of the comparator group. In a subsequent study, Emery et al. analyzed the outcomes of 817 patients with BMs based on their location in the brain (supratentorial, brainstem, or cerebellum) [20]. All of the patients in this study received SRS treatment, and a small number (9%) had previously undergone surgery to remove the tumor. This study included a relatively large and homogeneous population of patients. The researchers found that overall survival was significantly worse for patients with brainstem tumors when compared to those with supratentorial or cerebellar tumors [20]. However, in line with the results of the present analysis, where brainstem lesions were excluded from analysis, there was no significant difference in overall survival between patients with cerebellar tumors and those with supratentorial tumors [20]. These results suggest that the location of the tumor—infra- versus supratentorial (except brainstem lesions)—may not have an impact on patient outcomes, although more research is needed to fully understand the relationship between tumor location and patient outcomes. Further, it has to be mentioned that in addition to OS, local tumor control is an increasingly important outcome measure following surgery for BM [21]. With regard to radiation therapy and systemic therapies that have shown significant promise in controlling systemic disease and improving survival rates, local tumor control in the brain after neurosurgcial resection has become a crucial factor in maintaining the patients’ neurological and functional status [22]. Achieving complete resection of tumors and preventing local tumor recurrence can not only maintain or even improve neurological function and quality of life, but can also enhance the efficacy of systemic therapies by reducing the tumor burden in the CNS [22]. Therefore, in addition to OS as a main outcome measure as presented in the present study, follow-up studies might additionally consider local tumor control as an important outcome measure in surgery for BM, particularly in the era of evolving systemic oncological therapies. In addition, focal radiation therapy which is the most recommended adjuvant treatment after the surgical removal of BM might also contribute in the field of outcome measure dependent on tumor localization. This approach is favored over whole-brain radiation therapy due to its outstanding control of the disease and low side effect profile [9] and available data indicate that radiation therapy provides relief from symptoms in a comparable manner for supra and infratentorial BM localization [23].

In summary, the location of BM (supratentorial or infratentorial) has been a topic of research for decades, but there are conflicting results from various studies. It is important to note that infratentorial location refers to both the brainstem and cerebellum, which have different outcomes in terms of the accessibility to surgically remove the tumor. Similarly, it is challenging to accurately interpret studies that include patients with differing preoperative patient- and disease-related conditions which in turn do not allow to specifically analyze the impact of tumor location. Here, the authors intend to provide a homogenized cohort of patients based on a comparative matched pair analysis for important patient- and disease-related prognostic factors in BM surgery. Further multicenter analyses are needed to fully understand the relationship between tumor location and patient outcomes in different populations and settings.

### Limitations

The present study weakens from several other limitations in addition to the design of a retrospective analysis. First, available data reflect the experience of a single neuro-oncological specialized center only. Also, a possible selection bias must be cautioned in regard to the small sample size. Furthermore, this analysis was only neurosurgically driven and did not consider subgroup analysis regarding tumor types, course of disease, local irradiation, or systemic treatment. Indication for surgery for this consecutive group of patients surgically treated BM was made within a weekly interdisciplinary tumor board meeting. It is important to notice that within these meetings patients were from various medical centers. After having received surgical BM resection, patients were transferred to the original hospital in order to conduct postoperative oncological treatment. Against this backdrop, outcome assessment in terms of local tumor control rates and “neurological death” instead of death due to systemic disease burden was beyond the scope of this retrospective analysis. Nevertheless, these data represent interdisciplinary treatment decisions and might provide a more homogeneous patient population due to the aforementioned propensity score matching for known prognostic predictors in patients with surgically treated BM.

## Conclusions

The present study suggests that the prognostic value of infra- and supratentorial BMs does not significantly differ in patients that undergo surgery for solitary BM. These results might encourage physicians to induce surgical therapy of supra- and infratentorial BM in a similar manner.

## Data Availability

Restrictions apply to the availability of these data due to privacy restrictions.
